# A cross-sectional description of mobile food vendors and the foods they serve: potential partners in delivering healthier food-away-from-home choices

**DOI:** 10.1186/s12889-019-7075-8

**Published:** 2019-06-13

**Authors:** Melissa M. Reznar, Katherine Brennecke, Jamie Eathorne, Joel Gittelsohn

**Affiliations:** 1 Oakland University School of Health Sciences, 433 Meadow Brook Road, 3102 Human Health Building, Rochester, MI 48309 USA; 2 Johns Hopkins Bloomberg School of Public Health, 615 N. Wolfe Street, Room W2041, Baltimore, MD 21205 USA

**Keywords:** Mobile food vendors, Food trucks, Prepared foods, Foods away from home, Food environment, Obesity prevention, Food retail, Healthy foods, Restaurants

## Abstract

**Background:**

Food away from home (FAFH) in the US is associated with adverse health outcomes, and food dollars spent on FAFH continues to increase. FAFH studies have typically focused on restaurants and carryout establishments, but mobile food vendors – popularly known in the US as food trucks – have become more numerous and are an understudied segment of FAFH. The objective of this study was to assess mobile food vendors, their attitudes toward health and nutrition, and the foods they serve.

**Methods:**

This was a cross-sectional study of 41 mobile food vendors in Michigan, US. The survey contained questions about food and nutrition attitudes, such as barriers to putting healthy items on menus and perceived agreement with healthy food preparation practices. Participants were classified into a *healthy* and a *less healthy* attitude group based on whether they believed healthy menu items could be successful or not. In addition, participant menus were collected and analyzed according to whether menu items were healthy, moderately healthy, or unhealthy. Descriptive, univariate, and bivariate analyses were conducted.

**Results:**

Two-thirds of the participants felt that healthy menu items could be successful, and yet taste and value were the most important menu item success factors, each rated as important by 100% of the participants. Low consumer demand was the biggest barrier to putting healthy items on the menu (76%) whereas lack of chef interest (29%) and need for special training (24%) were the smallest. 72% of the vendors offered at least one healthy menu item, but only 20% of all reviewed menu items were healthy overall. There was no difference in the proportion of menu items that were healthy when comparing those with *healthy* attitudes (23% of menu items healthy) to those *less healthy* attitudes (17% of menu items healthy, *p* = 0.349).

**Conclusions:**

Mobile food vendors had positive views about putting healthy items on menus. However, a low proportion of menu items were classified as healthy. This suggests that mobile food vendors are promising potential public health partners in improving the health profile of FAFH, but that education of vendors is needed to ensure the success of healthier items.

**Electronic supplementary material:**

The online version of this article (10.1186/s12889-019-7075-8) contains supplementary material, which is available to authorized users.

## Background

Food away from home (FAFH) now accounts for nearly half of individual and family food expenditures in the United States, a figure that has risen steadily over time. In 1970, 26% of household food expenditures were for food consumed away from home compared to 44% in 2014 [1]. Although proportion of household FAFH food expenditures increases as household income increases, lower income households spend at least a third of their household food funds on FAFH [2]. While FAFH may offer convenience and perceived time savings, they tend to be higher in sodium, total fat, saturated fat, refined grains, and empty calories than foods prepared in the home [3–5]. High FAFH intake is associated with lower diet quality [6], higher BMI [7, 8], and presence of other cardiometabolic risk factors [9].

In the last 10 years, mobile food vendors, more popularly known in the US as food trucks, have dramatically increased, reaching an annual revenue of $960 million in 2017 and demonstrating an annual growth rate of 7.3%, outperforming most other sectors in food away from home [10]. Mobile food vendors have the advantage of much lower startup costs, lower overhead costs and the potential to travel, thereby optimizing convenience to customers. While market research data indicates that consumers are becoming more health conscious and that strategic mobile food vendors have increased the nutritional quality of their offerings [10], few studies in the nutrition and health literature have investigated mobile food vendors or their offerings, and their potential to be a point of public health intervention.

Two studies that have evaluated existing mobile food vendors have been identified [11, 12]. Both assessed vendors in different Latino immigrant communities and found that they tend to offer less healthy options, including processed snacks and meats and frozen desserts. Few of the mobile food vendors sold fruit and vegetables in precut or produce form (i.e. fruit or vegetables that requiring preparation, cutting, and/or peeling before eating), although the menus were not individually analyzed in either study [11, 12]. Conversely, there have been two intervention studies identified that have modified mobile food vendor menus and sought to promote healthier menu options, both of which reported some success [13, 14].

In the city of Detroit, there are over 956 full service and fast food restaurants, accounting for 27% of food outlets [15]. Yet, in their comprehensive assessment of Detroit’s food system, Taylor and Ard [15] did not include mobile food vendors, a potential point of public health intervention. Very few studies have featured interventions with mobile food vendors, despite their popularity coupled with their mobility, relatively low cost, and existing food preparation infrastructure. Moreover, there is a gap in the literature about mobile food vendors’ contribution to the FAFH as a whole. Ultimately, this knowledge can be used to improve the healthfulness of the food environment, and hopefully improve diet quality and lessen disease risk among consumers. Therefore, this study was conducted to answer three research questions about mobile food vendors in the Detroit Metropolitan area (Michigan, USA): (1) who are mobile food vendors? (2) what foods do they serve? and (3) what are mobile food vendors’ attitudes toward increasing healthful food preparation and menu offerings?

## Methods

### Procedures

A list of all known mobile food vendors in the Metropolitan Detroit area (Michigan, USA) was generated by Freedom of Information Act (FOIA) requests of licensing agencies, website searches, and in-person assessments. FOIA requests asking for names and addresses of all mobile food operators or food trucks, including those with Special Transitory Food Unit (STFU) licenses, were granted in four Detroit area counties and the city of Detroit. Website searches using internet search engines for “food trucks” and “mobile food” in various cities and throughout the Metropolitan Detroit area yielded mobile food vendor business websites, Facebook pages, Yelp profiles, and lists maintained by mobile food vendor booking agencies that provided additional mobile food vendors not identified by FOIA requests. Visits to events with multiple mobile food vendors also resulted in identification of additional mobile food vendors. Together, these activities produced a list of 75 mobile food vendors as of November 2016.

Mobile food vendors were contacted in November 2016 using a modified version of Dillman survey techniques [16]. Paper surveys were mailed to potential participants, along with a $5 pre-paid incentive. Approximately 6 weeks later, potential participants that had not responded on paper were contacted by email and invited to respond to an identical web-based version of the survey hosted on Qualtrics Survey Software (Qualtrics 2018, Provo, UT, available from www.qualtrics.com). All email and paper non-respondents were also messaged through Facebook Messenger and invited to participate in the web survey. Five paper surveys and five web surveys were received in response to the paper mailings and eight web surveys were received in response to email invitations. Facebook messaging did not generate any survey responses. Participants who opted to provide contact information received a $25 Amazon gift card or $21 Visa gift card (amount adjusted to account for service charges) upon completion of the survey.

In round one, distribution methods were not particularly successful, resulting in a low response rate (25%). First, mailing paper surveys were difficult, as 10 addresses were bad (those surveys were returned by mail), and we were unable to obtain mailing address for another 20 potential participants. Second, we learned that Facebook Messenger contact requests must be accepted by the recipients prior to then receiving and then reading the message in a multi-step process. No one contacted via Facebook Messenger was aware of or accepted our initial Facebook Messenger message delivery. Finally, one vendor was distrustful of the study’s motives and it was revealed after the conclusion of round one that the vendor had encouraged others – via mobile food vendor email listserv – not to participate. Because the Facebook Messenger process did not work as expected, prospective participants in round one would have been contacted by our team two times at most, provided that the paper mailings and emails were indeed received and opened by recipients. We did indicate that prospective participants could opt out of participation at any time and/or inform our research team to remove them from our contact list.

A second round of data collection was initiated in October 2017. The list of operating mobile food vendors was updated using web-based searching. This resulted in a list of mobile food vendors that were round one non-responders and were still operational at the start of round two as well as newer mobile food vendors that had never been contacted. In this round, mobile food vendors were mailed paper surveys, emailed invitations, and telephoned. Three responded to the mailed paper survey (one on paper, two on Qualtrics), 15 to the email, and 11 to the phone. In round two, 29 mobile food vendors responded (42% response rate). Combined, a total of 48 individuals responded, yielding 41 complete and usable surveys.

This study and all revisions in methodology were approved by the Institutional Review Board (IRB) at Oakland University. All participants either provided signed informed consent (paper survey), implied informed consent (online survey), or received an information sheet (paper survey post-amendment). The information sheet amendment (approved by the IRB) applied to those taking the survey on paper. Information sheets contained all the same information as the consent form, but the participants were not required to mail back a signed copy.

### Survey description

The survey consisted of 30 core questions and 12 demographic questions. Questions were grouped into three main categories: (1) attitudes toward health and nutrition of menus, (2) business operations, and (3) demographic information. Questions used in our survey were published in other works regarding chef attitudes toward menu nutrition and food truck operation. [17–21]. The compiled survey was reviewed by two experts in nutrition and survey development, one of whom is a PhD, RDN. After incorporating feedback from expert review, study personnel cognitively tested the survey with six individuals. The study team discussed cognitive testing feedback and modified questions as needed. The final survey used in this study is provided as an additional file [see Additional file 1].

In the attitudes section of the survey, one question asked participants to select three descriptors they believed to best *describe healthful meals* from a pre-defined list of 12 descriptors, including clean, fresh, good for you, and nutritious. Respondents could also write in their own descriptor(s). All other health attitude questions used four- or five-point Likert scales. One attitude question set asked participants to rate the *importance of factors to the success* (i.e. popularity) of food items – e.g. freshness, healthfulness, location of mobile food vendor, taste –from 1 = very unimportant to 5 = very important. Another set asked how *successful menu changes* like launching a new reduced/low calorie item or posting calorie content of foods on the menu would be on a scale from 1 = very unsuccessful to 5 = very successful. A third set asked about size of *barriers* (e.g. high ingredient cost, low consumer demand) to putting healthy options on the menu rated on a four-point scale from 1 = not a barrier at all to 4 = very big barrier. Mobile food vendors were also asked about *agreement with health statements* (e.g. preparing healthful food is costly and the food will not taste as good if it is healthy) and *agreement with importance of food preparation practices* (e.g. limiting use of processed foods, reducing the amount of salt in cooking) on a scale of 1 = strongly disagree to 5 = strongly agree as well as *frequency of food preparation practices* (e.g. reduce fat to cook food, use whole grains instead of refined flours) on a scale of 1 = never to 5 = always.

### Menu assessment

Menus from mobile food vendors were collected in various ways. Some mobile food vendors provided menus with full recipes for their items in exchange for nutrition analysis of those recipes. Other menus were obtained online or by visiting the mobile food vendor. Nutritionist Pro software version 5.3.0 (Axxya Systems, Stafford, TX) was used to analyze menu items. If mobile food vendors provided recipes, they were entered directly into Nutritionist Pro. When a recipe was not provided, standard menu items in Nutritionist Pro were used when possible (e.g. beef hot dog). If menu items were not included in the software database (e.g. beignets), five recipes were obtained online, and a representative recipe was chosen supported by pictures and observations of those menu items along with descriptions on the mobile food vendor’s menu. Menus were not acquired for five of the survey respondents because survey responses were submitted anonymously (*n* = 1), no menus were found online/vendor did not provide a menu (*n* = 2), menu items were not described specifically enough to allow for nutrition analysis (*n* = 1), and the vendor went out of business and no previous menu was available (*n* = 1).

To define menu items as healthy and less healthy for this study, US national recommendations were used as a template. The US Food and Drug Administration established a definition of healthy meal products and main dishes [22] to encourage healthier choices within a whole dietary pattern and has recently indicated suggested modifications to that definition [23] to be in line with the most recent 2015–2020 US Dietary Guidelines [24]. Although this definition does not include a limit on kilocalories, a limit was applied in this study similar to other research and agencies [25–27] to suggest portion control within an average 2000 kcal diet pattern. Therefore, a menu item was defined as healthy if kilocalories per serving was ≤600, saturated fat was ≤10% of total kilocalories, and the item contained ≥10% of daily value of at least one of the following: calcium, iron, fiber, protein, potassium, and/or vitamin D. All other items were classified as less healthy.

### Data analysis

Survey data were descriptively analyzed using univariate one-way frequencies and percentages for categorical variables and means for continuous variables. In order to evaluate differences in attitudes and characteristics among those favoring healthful offerings compared to those less favorable, respondents were categorized into two groups – those indicating that healthfulness of menu items was very or somewhat important to success of food items (*healthy* group) and those indicating healthfulness as neutral, somewhat unimportant, or very unimportant (*less healthy* group). Other attitude scales were likewise condensed into dichotomous categories by combining points in the Likert scales (i.e. somewhat/very successful versus neutral/somewhat/very unsuccessful; somewhat/very big barrier versus slight/not a barrier; agree/strongly agree versus neutral/strongly/disagree; and most of the time/very often/always versus never/rarely). Categorical differences in dichotomous attitudes comparing the healthy and less healthy groups were analyzed using bivariate non-parametric Fisher’s exact tests. Differences in percentage of healthy menu items between the *healthy* and *less healthy* group were tested using the Wilcoxon rank sums test for non-parametric distributions. A *p*-value of less than 0.05 was considered to be statistically significant. Due to the small sample size and exploratory nature of the study, *p*-values of 0.05–0.10 were also reported, in order to ensure that important potential points of consideration approaching significance were not overlooked. All data were analyzed using SAS software version 9.3 (SAS Institute Inc., Cary, NC).

## Results

### Mobile food vendor characteristics

Half of the vendors were male and most were white non-Hispanic (Table 1**)**. As a group, they were well-educated, 59% having a bachelor’s or higher degree. Mean age was 39.6 years. Participants had a considerable amount of experience in the food service industry (mean 14.2 years ±12.6; range 2–60 years). Reflecting the relative novelty of mobile food vendors in the food service industry, respondents reported operating their food trucks for an average of 3.5 years ±2.8 (range 0.5–16 years). Most respondents were the owners (*n* = 32; 78%). Many owners also reported filling other roles like chef (*n* = 6) or manager (*n* = 8). Six respondents classified themselves as managers (15%) and one as the executive chef (2%).

**Table 1:** Demographic characteristics of food truck operators (*n* = 41)

Age (years; mean + standard deviation, [range])	39.6 ± 9.8	[24–66]
Gender		
Male	21 (51%)	
Female	18 (44%)	
Prefer not to identify	2 (5%)	
Race		
White, non-Hispanic	29 (71%)	
Black, non-Hispanic	1 (2%)	
Asian	2 (5%)	
Other	3 (7%)	
Multi-race	5 (12%)	
Unknown	1 (2%)	
Education		
High school/GED	1 (2%)	
Some college/Associate’s degree	14 (34%)	
Bachelor’s degree	17 (41%)	
Post-graduate degree	7 (17%)	
Other	1 (2%)	
Unknown	1 (2%)	

### Foods that Mobile food vendors serve

Mobile food vendors reported serving a variety of foods: international (including Asian/world fusion, Caribbean, Indian, Mexican, Spanish, sushi): 15 (37%); American (including pizza): 9 (22%); meat-centered (including barbeque, chicken, burgers, hot dogs): 7 (17%); dessert (including donuts and ice cream): 4 (10%); vegetarian/vegan/mostly vegetarian: 3 (7%); and café fare (including coffee, bagels, smoothies, sandwiches): 3 (7%).

Table 2 shows the categorization of menu items by health classification. Offerings were predominately not healthy; only 27% of the menu items were categorized as healthy and hence 73% as less healthy. The category of menu items that were healthiest were sushi rolls (80% healthy); soup (57% healthy); mixed dish entrees (a mixture of grains, vegetables, and protein, e.g. Teriyaki Chicken Rice Bowls; 52% healthy); and vegetable/starchy sides (like beans, rice, Brussel sprouts; 47% healthy). Conversely, pizza (0% healthy); burgers (15% healthy); and tacos (17% healthy). Vegetables were offered in the form of sides, side salads, entrée salads, mixed dish entrees, and vegetable wraps. Twenty of the mobile food vendors offered vegetables in these forms (53 items), although items were only classified as healthy in 15 of those trucks (75%). No vendors offered whole fruit or pre-cut fruit cups. In addition, no whole wheat options were offered for dishes wherein that was a possibility.

**Table 2:** Menu items by healthfulness (*n* = 368 menu items among 36 mobile food vendors)

	Healthy	Less Healthy
Sushi	12 (80%)	3 (20%)
Soup	4 (57%)	3 (43%)
Entree Mixed Dish	11 (52%)	10 (48%)
Side Veg	7 (47%)	8 (53%)
Entree Salad	5 (38%)	8 (62%)
Snack Finger Food	13 (30%)	31 (70%)
Entree Pasta	2 (25%)	6 (75%)
Sandwich	14 (25%)	43 (75%)
Entree Protein	4 (24%)	13 (76%)
Sandwich - Hot Dog	11 (19%)	47 (81%)
Fries	2 (18%)	9 (82%)
Dessert	5 (18%)	23 (82%)
Taco	4 (17%)	19 (83%)
Sandwich - Burger	6 (15%)	35 (85%)
Pizza	0 (0%)	10 (100%)

Overall, 29 of the 36 mobile food vendors with menus (81%) had at least one healthy menu item. By type of cuisine served, vegetarian (59% of items healthy), deli (47%), and international (33%) vendors had the highest proportion of healthy items whereas dessert (8%), meat-centered (14%), and American cuisine (22%) vendors had the lowest amount of healthy items on their menus.

### Attitudes about healthful food preparation: univariate

Among listed descriptors of healthful meals, participants selected fresh (49%), nutritious (41%), and limited/no artificial ingredients (37%) most often. Terms that were selected least often to describe healthful meals include: clean (12%), good for you (12%), simple/few ingredients (12%), and contains certain food/components (10%).

Means of attitude scales among all participants and percentages of participants at the strong end of the attitude scales are displayed in Tables 3, 4, 5 and 6. Respondents rated taste as the most vital of success factors for menu items (Table 3), with 100% rating it as very or somewhat important to an item’s success, whereas healthfulness was indicated as very or somewhat important to success by 66% of respondents.

**Table 3:** Attitudes about important characteristics of menu items and potential of menu changes to be successful

	Mean	Std Dev	Median	Range	All (*n* = 41)	Healthy Group (*n* = 27)	Less Healthy Group (*n* = 14)	*p*-value
Importance of factors to popularity of menu items	5-point scale				% somewhat/very important			
Tastes great	4.9	0.3	5.0	(4–5)	100%	100%	100%	N/A
Good value	4.8	0.5	4.0	(4–5)	100%	100%	100%	N/A
Speed at which they are served	4.7	0.7	5.0	(3–5)	98%	100%	93%	0.342
Location of food truck	4.5	0.6	5.0	(3–5)	95%	93%	100%	0.539
Freshness^a1^	4.5	0.5	5.0	(2–5)	95%	100%	86%	0.117
Visual appeal of food^a1^	4.4	0.6	4.5	(3–5)	93%	96%	86%	0.276
Quality of ingredients^a1^	4.4	0.8	5.0	(2–5)	88%	100%	64%	0.003^b^
Time of day	4.1	0.8	4.0	(3–5)	73%	74%	71%	1.000
Healthfulness	3.6	0.8	4.0	(2–5)	66%	100%	0%	N/A
Potential of menu changes to be successful	5-point scale				% somewhat/very successful			
Launch new reduced or low calorie item	3.3	0.8	3.0	(2–5)	37%	48%	14%	0.044^b^
Reduce calorie content of some foods on the menu	3.1	0.9	3.0	(1–5)	32%	37%	21%	0.482
Make item reduced or low calorie	3.0	0.8	3.0	(1–5)	27%	26%	29%	1.000
Post calorie information for foods	2.8	1.0	3.0	(1–5)	22%	22%	21%	1.000
Reduce portion sizes of high calorie foods on the menu	2.6	0.9	3.0	(1–5)	12%	15%	7%	0.645

^a1^1 missing; ^a2^2 missing; ^a3^3 missing

^b^*p* < 0.05

^c^0.05 < = *p* < 0.1

**Table 4:** Perceived barriers to putting healthy options on the menu

	Mean	Std Dev	Median	Range	All (*n* = 41)	Healthy Group (*n* = 27)	Less Healthy Group (*n* = 14)	*p*-value
	4-point scale				% somewhat/very big			
Low consumer demand	3.0	1.0	3.0	(1–4)	76%	74%	79%	1.000
High ingredient cost	2.8	1.1	3.0	(1–4)	63%	63%	64%	1.000
Short ingredient shelf life	2.3	1.1	2.0	(1–4)	49%	52%	43%	0.744
High labor cost^a1^	2.3	1.0	2.0	(1–4)	43%	44%	38%	1.000
Too much time to cook/assemble food	2.2	1.2	2.0	(1–4)	39%	33%	50%	0.332
Limited ingredient availability	2.0	1.1	2.0	(1–4)	34%	37%	29%	0.734
Lack of chef interest in preparing healthy options	2.0	1.0	2.0	(1–4)	29%	33%	21%	0.494
Need for specific staff skills and training	1.7	0.9	1.0	(1–4)	24%	30%	14%	0.447

^a1^1 missing; ^a2^2 missing; ^a3^3 missing

^b^*p* < 0.05

^c^0.05 < = *p* < 0.1

**Table 5:** Agreement with statements about health and food preparation practices

	Mean	Std Dev	Median	Range	All (*n* = 41)	Healthy Group (*n* = 27)	Less Healthy Group (*n* = 14)	*p*-value
	5-point scale				% agree/strongly agree			
Agreement with health statements								
When served a large portion of food, it’s the customer’s responsibility to eat an appropriate amount.	3.5	1.1	4.0	(1–5)	61%	48%	86%	0.041^b^
The amount of food served at food trucks influences how much people eat	3.2	1.3	3.0	(1–5)	37%	30%	50%	0.306
Analyzing a recipe for nutrient content is a difficult task	3.2	1.0	3.0	(1–5)	46%	33%	71%	0.026^b^
Customers do not care about the healthfulness of menus	3.1	1.1	3.0	(1–5)	37%	30%	50%	0.306
Recipe modification is time consuming	3.1	1.0	3.0	(1–5)	32%	30%	36%	0.734
It is important to provide nutrition information for customers^a2^	2.9	1.1	3.0	(2–5)	31%	32%	29%	1.000
Preparing healthful food is costly^a2^	2.8	1.2	3.0	(1–5)	31%	27%	38%	0.486
It is not necessary for food trucks to provide healthful meal items	2.8	1.1	3.0	(1–5)	20%	11%	36%	0.097^c^
Chefs are not trained to cook healthfully^a1^	2.4	1.1	2.0	(1–5)	13%	15%	7%	0.640
The food will not taste as good if it is healthy	2.0	1.0	2.0	(1–4)	12%	11%	14%	1.000
Agreement with food preparation practices. “It is important to…”								
Limit use of processed foods	4.4	0.7	5.0	(1–5)	98%	96%	100%	1.000
Provide a vegetarian selection on menu	4.7	0.6	5.0	(2–5)	98%	100%	93%	0.342
Use more canola or olive oil vs vegetable or corn oil^a1^	4.2	0.8	4.0	(2–5)	83%	81%	86%	1.000
Provide more fruit and vegetables selection as part of menu offerings	4.1	0.8	4.0	(2–5)	76%	78%	71%	0.712
Reduce refined sugar in recipe preparation^a1^	4.0	0.8	4.0	(2–5)	75%	73%	79%	1.000
Use lean beef and pork, and trim the excess fat off poulty^a3^	3.6	1.0	4.0	(1–5)	63%	75%	43%	0.081^c^
Reduce the amount of salt in cooking	3.6	1.0	4.0	(1–5)	56%	63%	43%	0.322
Increase use of grains, rice, and legumes in meal preparation	3.6	0.9	4.0	(1–5)	51%	63%	29%	0.052^c^
Reduce fat content with the type of ingredient used	3.4	1.0	4.0	(1–5)	51%	63%	29%	0.052^c^
Substitute oil for butter in cooking^a2^	3.3	1.2	3.0	(1–5)	49%	60%	29%	0.096^c^

^a1^1 missing; ^a2^2 missing; ^a3^3 missing

^b^*p* < 0.05

^c^0.05 < = *p* < 0.1

**Table 6:** Frequency of food preparation practices

	Mean	Std Dev	Median	Range	All (*n* = 41)	Healthy Group (*n* = 27)	Less Healthy Group (*n* = 14)	*p*-value
	5-point scale				% most of the time/very often/always			
Add more fruit and vegetables to menu items	3.1	1.2	3.0	(1–5)	68%	78%	50%	0.089^c^
Reduce fat used to cook food	3.1	1.5	3.0	(1–5)	56%	59%	50%	0.742
Bake, broil, grill, or steam instead of frying or sauteeing^a1^	2.8	1.4	3.0	(1–5)	55%	54%	57%	1.000
Choose products lower in salt or sodium^a1^	2.8	1.2	2.5	(1–5)	50%	58%	36%	0.320
Reduce the amount of sugar or other sweeteners^a1^	2.7	1.2	2.5	(1–5)	50%	54%	43%	0.741
Use whole grains instead of refined flours^a1^	2.5	1.3	2.0	(1–5)	40%	42%	36%	0.746
Reduce the portion size of meat and substitute beans or grains	2.4	1.3	2.0	(1–5)	32%	37%	21%	0.482
Use vegetable, fruit, or starch purees to add moisture instead of fats	2.2	1.4	2.0	(1–5)	32%	37%	21%	0.482
Use fruit juices, broth, or other subs for oil in dressings and marinades^a1^	2.1	1.2	2.0	(1–5)	30%	42%	7%	0.030^b^
Use low-fat/nonfat milk or cheese instead of whole milk, cream, or cheese	1.6	0.9	1.0	(1–5)	7%	7%	7%	1.000

^a1^1 missing; ^a2^2 missing; ^a3^3 missing

^b^*p* < 0.05

^c^0.05 < = *p* < 0.1

Regarding potential success of menu changes in favor of health, mobile food vendors reported that launching a new item that is reduced/low calorie would be more successful than reducing the calorie content of foods already on the menu or reducing portion sizes of high calorie foods on the menu.

Low consumer demand was the biggest barrier to putting healthy options on the menu (Table 4) although high ingredient cost was another barrier rated to be somewhat/very big by a majority of the participants. Conversely, need for specific staff skills and lack of chef interest in healthy options were designated as somewhat or very big barriers by less than a third of the operators.

Among statements about health and nutrition (Table 5), a high proportion of participants agreed with the statement about customer responsibility to eat an appropriate amount when served a large portion. The lowest proportion agreed with food not tasting good if healthy and chefs not being trained to cook healthfully. Less than a third agreed that preparing healthy food is costly or that recipe modification is time consuming.

Regarding perceived importance of healthy food preparation practices, nearly all respondents indicated that it is important to provide a vegetarian selection on the menu to limit use of processed foods. In addition, using canola or olive oil in lieu of vegetable/corn oil, providing more fruit and vegetables on the menu, and reducing refined sugar were practices reported as important by more than three-quarters of the respondents. The lowest amount of agreement was with statements related to fat in cooking with only 49% agreeing or strongly agreeing it is important to substitute oil for butter and 51% with reducing fat content using low-fat ingredients.

In the set of self-reported food preparation practices (Table 6), a high percentage of mobile food vendors said that they often added more fruit and vegetables to menu items, more than reported reducing portion size of meat and substituting beans or grains. Use of low-fat dairy was not reported frequently.

### Attitudes about healthful food preparation: bivariate

Overall, 66% of the participants reported that healthfulness was a somewhat or very important factor to the success of menu items. This *healthy* group with positive attitudes about the potential success of healthy menu items was similar to the *less healthy* group in race/ethnicity, age, and education level. However, the *healthy* group was more likely to be female, with 56% of the *healthy* group reported to be female compared to 21% of the *less healthy* (*p* = 0.0435).

As Table 3 indicates, a significantly higher percentage of the *healthy* group also named quality of ingredients as an important factor compared to the *less healthy* group. The *healthy* group was also more likely to report that a new reduced or low calorie menu item would be somewhat/very successful. A similar proportion of *healthy* and *less healthy* operators thought reducing calorie content or changing an item to make it low or reduced calorie would be successful. There were no differences between the *healthy* and *less healthy* groups regarding barriers to putting healthy items on menus (Table 4).

A greater segment of the *less healthy* group responded that it is the customer’s responsibility to eat an appropriate amount of a large portion (Table 5) and that analyzing a recipe for nutrient content is difficult. In addition, more of the *less healthy* group indicated that it is not necessary for mobile food vendors to provide healthful meal items. There were no differences between groups about food not tasting good if healthy, with both groups demonstrating lack of agreement with the statement. The *healthy* group accorded a higher degree of importance to food preparation practices like using lean meats; increasing grains, rice, and legumes in meals; reducing fat content with ingredients; and substituting oil for butter. The *healthy* group reported adding fruit and vegetables to menu items (Table 6) and using fruit juice or broth as substitutes for oil more frequently than the *less healthy* group, suggesting that healthy attitudes and practices align regarding fruits and vegetables in cooking.

However, in examining the healthfulness of the mobile food vendor menu items, the *healthy* attitude was no more likely to serve healthy food options than the *less healthy* group. In the *healthy* group, 74% (17 of 23) served at least one healthy item compared to 69% of the *less healthy* group (9 of 13; *p* = 1.000). The percentage of the *healthy* group’s menu comprising healthy options was 23% compared to 17% of the *less healthy* group (*p* = 0.349).

## Discussion

This is the first study that has surveyed mobile food vendors to assess their demographic characteristics, types of food served, and their attitudes about healthfulness of menu items. Overall, two-thirds of the mobile food vendors indicated that healthful menu items could be successful, yet all other listed attributes were indicated as more important, especially taste and value. This is consistent with a nationally-representative survey showing that consumers place heavy emphasis on taste and cost [18]. Health, profit, and taste need not be mutually exclusive; mobile food vendors can marry those attributes by tailoring preferences of their clientele by venue. For instance, a mobile food vendor desiring to offer healthy foods in an after-school setting may serve pre-cut fruit to children opting for a quick, sweet snack whereas a mobile food vendor serving lunch entrees to adults may be more successful operationalizing healthfulness as grilled rather than fried meat along with a vegetable side or by offering healthy entrée salads. These healthier options need to be marketed wisely to be successful with customers. Similar to other studies, healthy was defined primarily by the terms fresh, nutritious, and limited/no artificial ingredients and processed foods [28], while another study showed restaurant executives yielded definitions primarily based on calories and fat content [29]. Several studies have noted the association of the word healthy with bad tasting food and preference for using terms such as fresh or in-season in marketing healthy items [29–31].

A variety of interventions can be employed to improve healthfulness of consumer choices, both covert (e.g. increasing healthful options or modifying existing items) and overt (e.g. displaying nutritional information or other messaging at point-of-purchase). The attitude toward such changes were not generally positive among respondents. This is likely tied to vendor food concept and corresponding equipment available on the truck. For instance, a vendor with a truck focused on fried chicken wings with deep fryers as the primary equipment on the truck would likely be hesitant to modify existing menu items. Launching a new reduced calorie item was rated most positively. Fewer were in favor of making an existing menu item lower in calories, consistent with other research about chef attitudes about healthy menu options [19], and indicating mobile food vendors may be hesitant to make changes that may disappoint regular customers and impact profit. Nonetheless, interventions that incorporate both point-of-purchase techniques and improved availability of healthful offerings – by introducing new options or modifying existing ones – have exhibited some success [32].

Respondents were particularly resistant to the idea of reducing portion sizes of high calorie food items, which parallels the emphasis that respondents put on value as an attribute of success of menu items. While some mobile food vendors recognized the amount of food served can influence how much people eat, respondents indicated it is the customer’s responsibility to eat an appropriate amount. Increased portion sizes have been consistently linked with increased caloric intake [33, 34]. Restaurants have indicated that large portion sizes help them compete by appealing to customers’ sense of economic value [21]. Other methods that enhance perceived value, like combo meals, customizable meal options, or portions offered in multiple sizes, may be more likely to be adopted by mobile food vendors than reducing portion size. Combination meal strategies have been successful in other FAFH settings [35].

Fat reduction was also not supported in beliefs or practices. Other food preparation practices that are not frequently used by mobile food vendors include sodium reduction, sugar reduction, and use of whole grains, which was reflected in food menu analysis. Importantly, no vendors offered fruit and few dishes with vegetables were healthy. However, given the results of the survey, it appears that making initial small menu changes like increasing the amount of vegetables and fruit relative to the remainder of a dish, offering fresh fruits and vegetables as side dishes, limiting or substituting fatty sauces/dressing, and offering baked and grilled options would be most acceptable to mobile food vendors and have been incorporated in prepared food settings [27, 36].

Mobile food vending respondents did not agree that healthy food will not taste good and that chefs are not interested in or trained to cook healthfully. Moreover, few participants indicated that healthy recipe modification is time consuming or costly. These findings suggest that chefs are interested in preparing healthy food, are equipped to do so, will prepare healthy options that taste good, and do not face time or cost barriers. Yet, about a third of the respondents agreed that customers do not care about health of menus, and indeed low consumer demand was the largest barrier to putting healthy options on menus. Techniques like taste testing new healthy menu items could potentially be employed to bridge the gap between chef interest/ability and perceived lack of customer demand [30, 37, 38]. Finding creative ways to market healthier items and work cooperatively with mobile food vendors may reduce mobile food vendor hesitance to change and strengthen partnerships [39–43]. One intervention study provided carryout owners with a limited stock of a healthy side and drink, supplied cooking equipment like grills, and equipped them with attractive menu boards highlighting healthier options [27]. These strategies were important for establishing rapport with the owners to support an intervention that was feasible, had high fidelity, and increased purchasing of healthy items by customers.

There was strong support for offering a vegetarian item on menus, with nearly all participants agreeing or strongly agreeing with the importance of doing so, and indeed, the menu items that tended to be healthy were primarily plant-based. The presence of vegetarian items is likely a direct response to consumer demand, as agencies like the National Restaurant Association report high demand for vegetarian/vegan items, vegetable-forward cuisine, vegetable carb substitutes, plant-based burgers, and fruit and vegetables as sides for kid’s meals when dining out [44].

This study does have some limitations. The sample size is small and response rate was low. Considerable effort was extended to maximize response rate by using two rounds of multi-modal contact. Our response rate of 42% in round two was consistent with average response rates of multimodal studies, which typically average 35 to 60%, depending on target population and distribution techniques used [45–47]. In addition, response bias was possible in that respondents to this survey may have been more interested in health and nutrition than the general population of mobile food vendors. We only included mobile food vendors based in Michigan, USA, and so their attitudes and practices may differ from those elsewhere. Education level of respondents in this study was higher than that of US business owners in the accommodation and food service industry [48]. Thus, the results may not necessarily represent the overall education level of all mobile food vendors. Consumer-related attributes, such as mobile food vendors’ target audiences and venue of mobile food vendor operation (e.g. food truck festival, lunch venue), were not included in this study. Finally, given that we did not receive recipes from all mobile food vendors, some food items may have been misclassified as healthy or unhealthy.

Mobile food vendors may be one potential unexplored avenue to increase diet quality of consumers away from home. Although mobile food vendors do not perceive healthfulness to be a particularly important selling feature, many are neutral or open to the importance of selling healthy food to their customers. Value placed on reduction in processed foods, increasing fruit and vegetables in menu items, and offering vegetarian menu items along with the perception that they can prepare healthy food that tastes good indicate strong potential to improve healthfulness of menu offerings.

Most importantly, mobile food vendors are exciting partners. Even though they are small segments of the overall food scene, their mobility allows them to reach many and various types of customers. If a vendor serves 80 customers an hour, it can potentially reach 400 people in a meal rush. That same food truck might go to an entirely different venue and population on a different day to reach an entirely different population. Seasonality is a limitation of mobile food vendors in four-season climates, but they often serve as catering trucks to customers in enclosed venues in the winter months. This extends customer reach year-round. If coupled with consumer education, partnership with mobile food vendors can be a meaningful way to positively influence many people.

## Conclusions

Mobile food vendors report positive attitudes about the potential success of healthy menu items, and many mobile food vendors did offer at least one healthy menu item. Moreover, they indicate that they have interest in doing so and do not need additional training. However, most of the menu items overall were unhealthy, indicating that there is room for growth in the number of healthy items as well as education of mobile food vendors about nutritional criteria of healthy menu items. Taste-testing is one way that chefs and owners can showcase that they, in partnership with dietitians and nutritionists, are able to prepare healthy foods that taste great.

Mobile food vendors do not offer fruit and offer few healthy items with vegetables. Vendors were also resistant to reducing portion size. Vendors were most amenable to offering fruits and vegetables as sides or to incorporate them into dishes. In fact, a high proportion acknowledged the importance of providing a vegetarian menu item. Offering new menu items coupled with fruit/vegetable sides or with a high proportion of mixed in fruits and/or vegetables may be an ideal first menu modification strategy to try when beginning work with mobile food vendors, as they were more willing to create new healthier menu items than to modify existing ones. In addition, vendors can try strategies like combo meals, multiple sizes of items, or ala carte items to enhance the perceived value while reducing overall portion size. It will be important to market such options wisely, as vendors indicate that the adjective “healthy” tends to typically turns off customers. Findings suggest that phrases like fresh and nutritious may be more enticing to consumers.

However, the best indicator of mobile food vendor purchasing is consumer themselves. Therefore, the next important stage of this research is to survey customers, including customers’ reasons for visiting vendors, how often they frequent or would frequent vendors, what items they typically purchase, and their views on health and nutrition. Future directions of our research include consumer intercept surveys to elicit this information. Similarly, examining mobile food vendor sales data to identify items that do and do not sell well, indicating consumer preferences, is also planned.

Overall, mobile food vendors are potential partners in the service of public health, by providing tasty, healthy prepared food items in a variety of settings like after-school programs, summer adult and child recreation and community centers, and outdoor health fairs. These activities pave the way for interventions with mobile food vendors to improve the health profile of the FAFH food environment and diet quality of consumers. Ultimately, such endeavors will contribute to other efforts to reduce obesity and other related adverse health outcomes.

## Additional file

Additional file 1: Food Truck Research Study. Complete final survey given to study participants. (PDF 340 kb)

## Data Availability

The datasets generated and analyzed during the current study are not publicly available because they contain identifiable information. Sharing research data would compromise individual privacy.

## Abbreviation

FAFH: Food away from home
