# Supplementary material for: A cross-sectional description of mobile food vendors and the foods they serve: potential partners in delivering healthier food-away-from-home choices
Source: BMC Public Health. 2019 Jun 13;19:744. doi: 10.1186/s12889-019-7075-8 (PMC6567609; doi:10.1186/s12889-019-7075-8)
Supplement: Supplementary file 1 — Food Truck Research Study. Complete final survey given to study participants. (PDF 340 kb) [file 12889_2019_7075_MOESM1_ESM.pdf]

OAKLAND UNIVERSITY

Food Truck Research Study

A research study of food truck operators in Metro Detroit

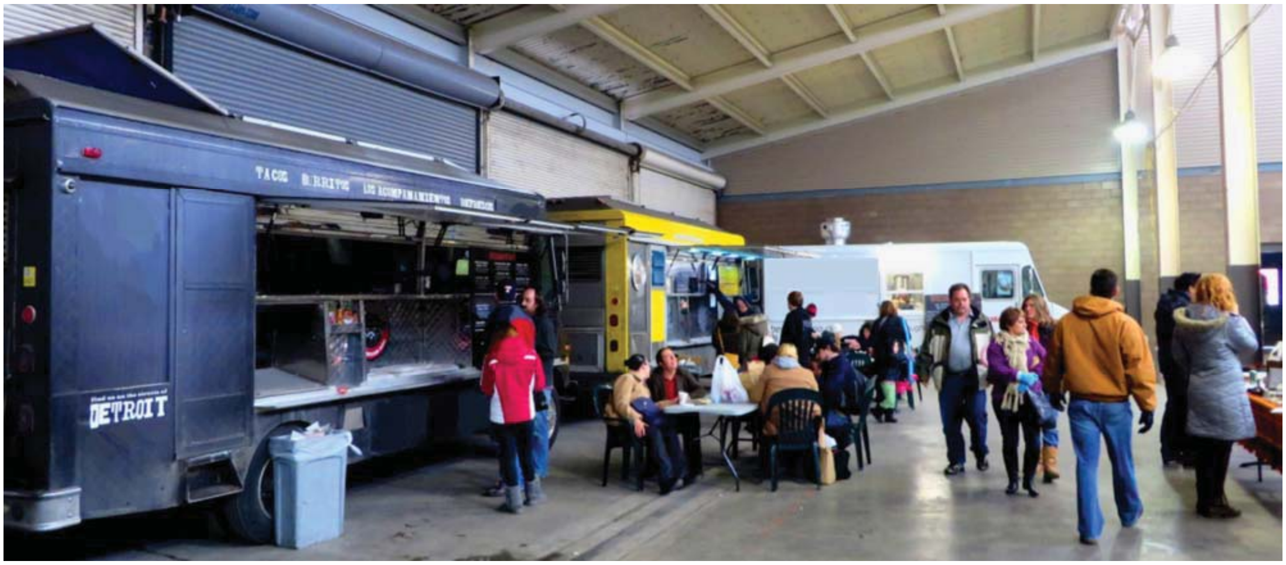

School of Health Sciences
3102 Human Health Building
433 Meadow Brook Road
Rochester, MI 48309

To be completed by an active mobile food truck operating in Michigan.

SECTION 1. ABOUT MENUS

Please tell us a little about your opinion on menus, customer decision-making, and food preparation practices.

Which 3 terms best describe healthful meals? Please select only 3.

- ☐ Clean
- ☐ Contains certain food/components
- ☐ Does not contain (or has low levels of) certain components (e.g. low fat, low sugar, low calorie)
- ☐ Fresh
- ☐ Good for you
- ☐ Limited or no artificial ingredients or additives
- ☐ Natural
- ☐ Non-GMO
- ☐ Nutritious
- ☐ Unprocessed/unadulterated
- ☐ Organic
- ☐ Simple/few ingredients
- ☐ Other ____________

Rate how important or unimportant you think the following factors are for success, in terms of popularity, of food items at food trucks.

	Very Important	Somewhat Important	Neither Important Nor Unimportant	Somewhat Unimportant	Very Unimportant
Freshness	○	○	○	○	○
Good value	○	○	○	○	○
Healthfulness	○	○	○	○	○
Location of food truck	○	○	○	○	○
Quality of ingredients	○	○	○	○	○
Speed at which they are served	○	○	○	○	○
Tastes great	○	○	○	○	○
Time of Day	○	○	○	○	○
Visual appeal of food	○	○	○	○	○

Rate how important or unimportant you think the following factors are for success, in terms of popularity, of food items at food trucks.

	Very Important	Somewhat Important	Neither Important Nor Unimportant	Somewhat Unimportant	Very Unimportant
Freshness	O	O	O	O	O
Good value	O	O	O	O	O
Healthfulness	O	O	O	O	O
Location of food truck	O	O	O	O	O
Quality of ingredients	O	O	O	O	O
Speed at which they are served	O	O	O	O	O
Tastes great	O	O	O	O	O
Time of Day	O	O	O	O	O
Visual appeal of food	O	O	O	O	O

Rate how successful, <u>in terms of sales,</u> you think each of the following menu changes would be.

	Very Successful	Somewhat Successful	Neither Successful Nor Unsuccessful	Somewhat Unsuccessful	Very Unsuccessful
Make an existing menu item reduced / low-calorie	O	O	O	O	O
Launch a new menu item that is reduced/low-calorie	O	O	O	O	O
Post calorie information for all foods on the menu	O	O	O	O	O
Reduce the calorie content of some foods on the menu	O	O	O	O	O
Reduce the portion sizes of high calorie foods on the menu	O	O	O	O	O

Please indicate the extent to which the following items are barriers to putting healthy options on your restaurant's menu. Be sure to rate each item.

	Very big barrier	Somewhat of a barrier	Slight barrier	Not a barrier at all
High ingredient cost	O	O	O	O
High labor cost	O	O	O	O
Lack of chef interest in preparing healthy options	O	O	O	O
Limited ingredient availability	O	O	O	O
Low consumer demand	O	O	O	O
Need for specific staff skills and training	O	O	O	O
Short ingredient shelf life	O	O	O	O
Too much time to cook /assemble food	O	O	O	O

How much do you agree or disagree with each of the following statements about healthfulness of food items?

	Strongly agree	Agree	Neither agree nor disagree	Disagree	Strongly disagree
Analyzing a recipe for nutrient content is a difficult task.	O	O	O	O	O
Chefs are not trained to cook healthfully.	O	O	O	O	O
Customers do not care about the healthfulness or nutrient content of food truck menus.	O	O	O	O	O
It is important to provide nutrition information for customers.	O	O	O	O	O
It is not necessary for food trucks to provide healthful meal items.	O	O	O	O	O
Preparing healthful food is costly.	O	O	O	O	O
Recipe modification is time consuming.	O	O	O	O	O
The amount of food served at food trucks influences how much people eat.	O	O	O	O	O
The food will not taste as good if it is healthy.	O	O	O	O	O
When served a large portion of food, it is the customer's responsibility to eat an appropriate amount.	O	O	O	O	O

How often do you (or your chef if you do not do most of the cooking) use each of the following food preparation practices?

	Always	Very often	Most of the time	Rarely	Never
Add more fruit and vegetables to menu items	○	○	○	○	○
Bake, broil, grill, or steam instead of frying or sautéing	○	○	○	○	○
Choose products lower in salt or sodium	○	○	○	○	○
Reduce fat used to cook food	○	○	○	○	○
Reduce the amount of sugar or other sweeteners in recipes	○	○	○	○	○
Reduced the portion size of meat and substitute beans or grains	○	○	○	○	○
Use low-fat / nonfat milk or cheese instead of whole milk, cream, or cheese	○	○	○	○	○
Use whole grains instead of refined flours	○	○	○	○	○
Use vegetable, fruit, or starch purees to add moisture instead of fats	○	○	○	○	○
Use fruit juices, broth, or other substitutes for oil in dressings and marinades	○	○	○	○	○

How much do you agree or disagree with each of the following statements?

It is important to ...

	Strongly agree	Agree	Neither agree nor disagree	Disagree	Strongly disagree
Increase the use of grains, rice, and legumes in meal preparation?	O	O	O	O	O
Limit the use of processed foods?	O	O	O	O	O
Provide a vegetarian selection on the menu?	O	O	O	O	O
Provide more fruit and vegetable selections as part of menu offerings?	O	O	O	O	O
Reduce fat content with the type of ingredient used?	O	O	O	O	O
Reduce refined sugar in recipe preparation?	O	O	O	O	O
Reduce the amount of salt in cooking?	O	O	O	O	O
Substitute oil for butter in cooking?	O	O	O	O	O
Use lean beef and pork, and trim the excess fat off poultry?	O	O	O	O	O
Use more canola or olive oil vs vegetable or corn oil?	O	O	O	O	O

How familiar are you with the calorie content of the items on your menu?

- Extremely familiar
- Very familiar
- Somewhat familiar
- Not very familiar
- Not at all familiar

What is your interest level in completing a comprehensive analysis of the nutrition content (including calorie, fat, sodium, carbohydrate, and more information) of the foods you serve? (Select the best response)

- Very interested
- Somewhat interested
- Neither interested or uninterested
- Somewhat uninterested
- Very uninterested
- I already completed a comprehensive nutrition analysis

SECTION 2. ABOUT YOUR FOOD TRUCK

We are interested in understanding how the costs and operation of a food truck influence practices and choices concerning healthy eating.

In addition, we are considering whether food trucks would be a viable option as an university course. Therefore, we would like to better understand mobile food truck economics and business.

How long has your food truck been in operation (years)?

How long have you been in the food service industry? Please consider all food service experience.

What communities / areas do you regularly serve? (select all that apply)

- ☐ Ann Arbor
- ☐ Birmingham
- ☐ Berkley
- ☐ Detroit-Campus Martius
- ☐ Detroit-Eastern Market
- ☐ Detroit-WSU Campus
- ☐ Ferndale
- ☐ Royal Oak
- ☐ Troy
- ☐ Other (please describe)

How would you characterize the type of cuisine you serve? (select the best answer)

- O American
- O Bagel / Deli
- O BBQ
- O Burgers
- O Chicken
- O Chinese
- O Coffee
- O Comfort Food
- O Donut / Bakery / Pastry
- O French
- O Greek, Middle Eastern
- O Ice Cream / Frozen Yogurt
- O Indian
- O Italian
- O Japanese
- O Mexican
- O Pizza
- O Seafood
- O Steakhouse
- O Sub Sandwiches
- O Thai
- O Vegetarian
- O Other (please describe)

What type of vehicle do you operate out of?

- Mobile truck with full kitchen
- Mobile truck without full kitchen
- Food cart
- Food trailer with full kitchen
- Food trailer without full kitchen

What was the purchase cost of the truck?

Did you complete any repairs or remodel the truck before beginning operations? ***If yes, please describe the repairs and remodels and their estimated costs in the chart below***

- Yes
- No

Cost	Repair/Remodel Description
$	
$	
$	
$	
$	

Please select the months of operation of your food truck. (Select <u>all</u> that apply)

Jan	Feb	Mar	Apr	May	Jun	Jul	Aug	Sep	Oct	Nov	Dec
☐	☐	☐	☐	☐	☐	☐	☐	☐	☐	☐	☐

Please select hours of the day *your food truck* operates for each day of the week during month operation. (Select <u>all</u> that apply)

	8 am or earlier	8-10 am	10 am-12 pm	12-2 pm	2-4 pm	4-6 pm	6-8 pm	8-10 pm	10 pm or later
Monday	☐	☐	☐	☐	☐	☐	☐	☐	☐
Tuesday	☐	☐	☐	☐	☐	☐	☐	☐	☐
Wednesday	☐	☐	☐	☐	☐	☐	☐	☐	☐
Thursday	☐	☐	☐	☐	☐	☐	☐	☐	☐
Friday	☐	☐	☐	☐	☐	☐	☐	☐	☐
Saturday	☐	☐	☐	☐	☐	☐	☐	☐	☐
Sunday	☐	☐	☐	☐	☐	☐	☐	☐	☐

Please select hours of the day *with the highest customer traffic* for each day of the week. (Select <u>all</u> that apply)

	8 am or earlier	8-10 am	10 am-12 pm	12-2 pm	2-4 pm	4-6 pm	6-8 pm	8-10 pm	10 pm or later
Monday	☐	☐	☐	☐	☐	☐	☐	☐	☐
Tuesday	☐	☐	☐	☐	☐	☐	☐	☐	☐
Wednesday	☐	☐	☐	☐	☐	☐	☐	☐	☐
Thursday	☐	☐	☐	☐	☐	☐	☐	☐	☐
Friday	☐	☐	☐	☐	☐	☐	☐	☐	☐
Saturday	☐	☐	☐	☐	☐	☐	☐	☐	☐
Sunday	☐	☐	☐	☐	☐	☐	☐	☐	☐

During months when your food truck <u>is in operation</u>, how many hours per week on average do you estimate are spent *working on any aspect* related to your mobile food business? (Select <u>the</u> best estimate)

Less than 10 hours per week	10-20 hours per week	20-30 hours per week	40-50 hours per week	50-60 hours per week	60-70 hours per week	70-80 hours per week	More than 80 hours per week
O	O	O	O	O	O	O	O

During months when your food truck <u>is not in operation</u>, how many hours per week on average do you estimate are spent *working on any aspect* related to your mobile food business? (Select <u>the</u> best estimate)

Less than 10 hours per week	10-20 hours per week	20-30 hours per week	40-50 hours per week	50-60 hours per week	60-70 hours per week	70-80 hours per week	More than 80 hours per week
O	O	O	O	O	O	O	O

During months when your food truck <u>is in operation</u>, how many hours per week on average do you estimate is your *food truck open for food sales*? (Select <u>the</u> best estimate)

Less than 10 hours per week	10-20 hours per week	20-30 hours per week	40-50 hours per week	50-60 hours per week	60-70 hours per week	70-80 hours per week	More than 80 hours per week
O	O	O	O	O	O	O	O

How many customers do you serve a month, on average? (Select <u>the</u> best estimate)

Less than 50 customers	50-100 customers	100-200 customers	200-300 customers	300-400 customers	400-500 customers	More than 500 customers
O	O	O	O	O	O	O

Please select the titles or roles of all the members of your mobile food business team (including yourself). ***If any roles aren't listed below, please list them under "other." If one person perform multiple roles please describe in the "other" box below.***

- ☐ Owner
- ☐ Mobile food business manager
- ☐ Window attendant
- ☐ Kitchen Workers
- ☐ Chef (head cook, usually picks the menu for the day and has formal education/certification in cooking)
- ☐ Cook
- ☐ Driver
- ☐ Other

What is your average monthly estimate of the following while in operation:

$ ______ Gas Costs?

$ ______ Truck upkeep costs?

$ ______ Food / Ingredients?

What is your annual estimate of:

$ ______ Gas Costs?

$ ______ Truck upkeep costs?

$ ______ Food / Ingredients?

Which of the following social media outlets do you use to promote your business? (Select all that apply)

- ☐ Facebook
- ☐ Twitter
- ☐ Instagram
- ☐ Foursquare
- ☐ Custom website for your business
- ☐ Other (Please describe)
- ☐ I do not use any social media to promote my business

Through which social media avenues do you publicize each of the following?

	Facebook	Twitter	Foursquaure	Instagram	Business Website	Other Site	I do not publicize this
Location of truck	☐	☐	☐	☐	☐	☐	☐
Calendar of events	☐	☐	☐	☐	☐	☐	☐
Menu	☐	☐	☐	☐	☐	☐	☐
Pricing	☐	☐	☐	☐	☐	☐	☐
Nutritional Information	☐	☐	☐	☐	☐	☐	☐
Other	☐	☐	☐	☐	☐	☐	☐

SECTION 3. ABOUT YOU

Please tell us about you and your background.

What is your age (years)?

[ ]

What is your gender?

- ○ Male
- ○ Female
- ○ I prefer not to identify

How do you usually describe yourself? (select all that apply)

- ☐ White
- ☐ Black or African American
- ☐ Hispanic or Latino / a
- ☐ Asian or Pacific Islander
- ☐ American Indian, Alaskan Native, or Native Hawaiian
- ☐ Other

[ ]

What is the highest level of school you completed?

- Some high school
- High school diploma or GED
- Some college
- Associate degree
- Bachelor's degree
- Post-Graduate degree
- Other (please describe)

THANK YOU FOR COMPLETING OUR SURVEY!!!

Please complete the final details below.

Would you be interested in participating in an in-person interview to discuss these topics more in-depth? There will be a $50 incentive provided for those who decide to participate.

- Yes
- No

For your time and inconvenience, we would like to give you $25. Which of the following ways would you prefer to receive that thank you gift?

- $25 Amazon electronic gift card
- $21 Visa card (can be used at any store, amount reduced due to service fee)

If willing, please provide your contact information below so that we do not continue to contact you after you have completed the survey and to ensure smooth delivery of your $25 incentive. All information will be kept confidential.

Your name

Your role(s) or title(s)

Food truck name

Email address

Business address

Phone number

Please share any thoughts, comments, or concerns about the survey.

If you are interested in having us conduct a **nutritional analysis** of your menu items, using the enclosed return envelope, please send a **menu and detailed ingredient list** with quantities for items you sell. My contact information is as follows:

Melissa M. Reznar, PhD, MPH
Assistant Professor of Health Sciences
3102 Human Health Building
433 Meadow Brook Rd.
Rochester MI 48309-4452
(248) 364-8668
reznar@oakland.edu

If you are not interested in a nutritional analysis, but are willing to share a **menu and/or nutritional guide** for your food truck, please let us know. Thank you so much for taking the time to complete this survey! We truly appreciate your assistance in providing this information.
